# Biological Values of Acupuncture and Chinese Herbal Medicine: Impact on the Life Science

**DOI:** 10.1155/2014/593921

**Published:** 2014-01-12

**Authors:** Yong-Qing Yang, Chen Yan, Chris J. Branford-White, Xiang-Yu Hou

**Affiliations:** ^1^Shanghai Research Institute of Acupuncture and Meridian, Yueyang Hospital, Shanghai University of Traditional Chinese Medicine, Shanghai 201203, China; ^2^Cardiovascular Research Institute, University of Rochester School of Medicine and Dentistry, Rochester, NY 14642, USA; ^3^Institute for Health Research and Policy, London Metropolitan University, UK; ^4^School of Public Health, Queensland University of Technology, Victoria Park Road, Kelvin Grove, QLD 4059, Australia

As treasures of the traditional Chinese medicine (TCM), acupuncture and Chinese herbal medicine have a history of more than 2,500 years and have achieved sound effects in the clinical practice [1]. The effects of acupuncture and Chinese herbs have usually been demonstrated by biological regulations of physiological and pathological processes [2, 3], which are inherent responses of human beings and of great importance in the life science research.

In recent years, the researches of acupuncture and Chinese herbal medicine have improved significantly due to the prosperity and development of modern life science technology [4–7]. At the same time, the studies of acupuncture and Chinese herbal medicine have in turn enhanced the development of biomedical science as well as the understanding of life [8–10] and offered new perspectives that can benefit modern medicine [11, 12]. However, the major challenge of the integration of TCM with modern medicine is the inadequate understanding of the biological foundation of TCM experience and concepts. In order to deepen our knowledge of TCM, it is important to clarify the biological value and mechanistic action of acupuncture and Chinese herbal medicine, which may turn hypothesis to solid data-based research and creative discovery of life science.

We are excited to present our readers with this special issue that summarizes recent discoveries and knowledge of acupuncture and Chinese herbal medicine. This specialized issue collects 24 original research articles and 4 reviews that provide the clinical or animal-based evidence elucidating the impacts of acupuncture and Chinese herbal medicine on the life science. Among these original research articles, the biological functions and potential mechanisms of acupuncture and Chinese herbal drugs in disease treatment were demonstrated in animal models of diseases such as focal cerebral ischemia, pain, atopic dermatitis, insomnia, fatty liver, and Crohn's disease. Two papers are on cancer cells: Han-Peng Kuo et al. reported that Ganoderma tsugae extract inhibited the growth of HER2-overexpressing cancer cells and enhanced the growth inhibitory effect of antitumor drugs via the modulation of HER2/PI3K/Akt signaling pathway; Xiu-Feng Wang et al. showed that PC-SPESII herbal extract could impair human breast cancer metastasis by regulating proteolytic enzymes and matrix dynamics through the p38MAPK and SAPK/JNK pathway. Additionally, You Ning et al. reported the molecular mechanisms underlying the antiproliferative and prodifferentiative effects of psoralen on adult neural stem cells using DNA microarray. Interestingly, two research articles (Lei-Miao Yin et al. and Xiang-Gen Zhong et al.) simultaneously focused on the same issue, specific link between the lung and the large intestine, which provide new lines of evidence for the modern biological theory of “exterior-interior correlation between *Zang* and *Fu* organs.” The review by Yu Wang et al. presented a comprehensive overview of the researches on acupuncture effective biomolecules, covered diverse carriers such as cerebrospinal fluid, serum, organs, and tissues, and discussed how to promote the development of biological medicine based on the discovery of acupuncture effective biomolecules. The other 3 review articles also provided us with current understanding of biological values of acupuncture and Chinese herbal medicine on inflammatory bowel diseases, amblyopia, and glucose and lipids metabolism, respectively.

In summary, this special issue provides up-to-date and valuable information about the role of acupuncture and Chinese herbs in the life science. Accordingly, future researches with modern technologies would further advance our understanding of the biological effects and cellular/molecular bases for acupuncture and Chinese herbal medicine in diverse diseases.
